# Characterization of Bioactive Recombinant Human Lysozyme Expressed in Milk of Cloned Transgenic Cattle

**DOI:** 10.1371/journal.pone.0017593

**Published:** 2011-03-16

**Authors:** Bin Yang, Jianwu Wang, Bo Tang, Yufang Liu, Chengdong Guo, Penghua Yang, Tian Yu, Rong Li, Jianmin Zhao, Lei Zhang, Yunping Dai, Ning Li

**Affiliations:** 1 State Key Laboratory for Agrobiotechnology, China Agricultural University, Beijing, People's Republic of China; 2 Beijing GenProtein Biotechnology Company, Beijing, People's Republic of China; University of South Florida College of Medicine, United States of America

## Abstract

**Background:**

There is great potential for using transgenic technology to improve the quality of cow milk and to produce biopharmaceuticals within the mammary gland. Lysozyme, a bactericidal protein that protects human infants from microbial infections, is highly expressed in human milk but is found in only trace amounts in cow milk.

**Methodology/Principal Findings:**

We have produced 17 healthy cloned cattle expressing recombinant human lysozyme using somatic cell nuclear transfer. In this study, we just focus on four transgenic cattle which were natural lactation. The expression level of the recombinant lysozyme was up to 25.96 mg/L, as measured by radioimmunoassay. Purified recombinant human lysozyme showed the same physicochemical properties, such as molecular mass and bacterial lysis, as its natural counterpart. Moreover, both recombinant and natural lysozyme had similar conditions for reactivity as well as for pH and temperature stability during *in vitro* simulations. The gross composition of transgenic and non-transgenic milk, including levels of lactose, total protein, total fat, and total solids were not found significant differences.

**Conclusions/Significance:**

Thus, our study not only describes transgenic cattle whose milk offers the similar nutritional benefits as human milk but also reports techniques that could be further refined for production of active human lysozyme on a large scale.

## Introduction

Lysozyme, also known as muramidase, was first described by Alexander Fleming [1]. This enzyme is a type of glycanhydrolase, which hydrolyzes the β-1,4-linkages between N-acetylmuramic acid and N-acetyl-D-glucosamine residues in the peptidoglycan of bacterial cell walls. Lysozyme has been found in variety of species [2].

Human lysozyme (HLZ) is a C-type lysozyme that consists of a single polypeptide of 130 amino acid residues (molecular mass ≈14.7 kDa) [3]. It is a positively charged protein with high pI (≈11) under normal human physiological conditions [4]. HLZ is widely distributed in human tissues and body fluids (tears, saliva, milk) [5], [6] and it plays important roles as a non-specific immune factor and anti-inflammatory factor [7]. Furthermore, some reports have shown that HLZ has anti-fungal and anti-viral activities [8], [9]. Moreover, changes in the HLZ concentration in serum or urine is used as a diagnostic marker for certain diseases [10]. Also, HLZ is under study as a potentially useful material for use in food products, cosmetics (as a preservative), medicine feed, baby formula, and so on [11]–[13].

The benefits of lysozyme present in breast milk to improve immunity and prevent infection in infants, are gaining attention. It increases the levels of beneficial intestinal microflora and strengthens disease resistance in infants. These effects are believed to occur through the lysis of certain potentially damaging Gram-positive bacteria and a few Gram-negative bacteria in the gastrointestinal tract of breast-fed babies [14], [15]. The content of lysozyme in human milk ranges from 3 to 3000 µg/ml, and the typical concentration is about 200–400 µg/ml [16], [17]; however, only trace amounts are found in the breast milk of ruminants. Bovine milk typically contains only 0.05–0.22 µg/ml of lysozyme [16], [18]. In addition, its activity is 1/10 of lysozyme from human breast milk [16], [19]. Despite the benefits that HLZ provides to breast-fed infants, mothers do not always desire to lactate and sometimes situations prevent lactation; therefore, the development of alternate sources of HLZ would be beneficial to infant health.

The development of genetic engineering has enabled the expression of HLZ in microorganisms [20], eukaryotic cells [21] and plants [22]. In recent years, the mammary gland has been considered as a potential bioreactor for the expression of recombinant proteins [23], which appears to be capable of appropriate post-translational modifications of recombinant proteins [24]. After synthesis in mammary epithelial cells, recombinant proteins are immediately secreted into milk through the signal peptide design to the vector; this makes it easier to purify recombinant proteins using relatively simple chromatographic methods. Still, the milk of dairy cows is easily obtained and continuously available. So, using of the mammary gland bioreactor system of dairy cows provides not only a good new way to produce rHLZ but also a way to transfer the benefits of human milk to cow milk. Moreover, expression of rHLZ might help dairy animals resist the growth of bacteria which cause mastitis [25]. Maga et al. expressed rHLZ in the mammary gland of transgenic mice [26]. Shortly thereafter, a line of transgenic goats that expressed rHLZ was generated [27].

We previously produced transgenic mice that expressed rHLZ [28]. In the present study, we produced cloned transgenic cattle that expressed rHLZ in breast milk, and we tested the physicochemical characteristics of the rHLZ that was expressed. We also optimized a method for purifying rHLZ from breast milk of transgenic cattle for potential large-scale production in the future.

## Results

### Generation of cloned transgenic cattle that express rHLZ

**Figure 1:**
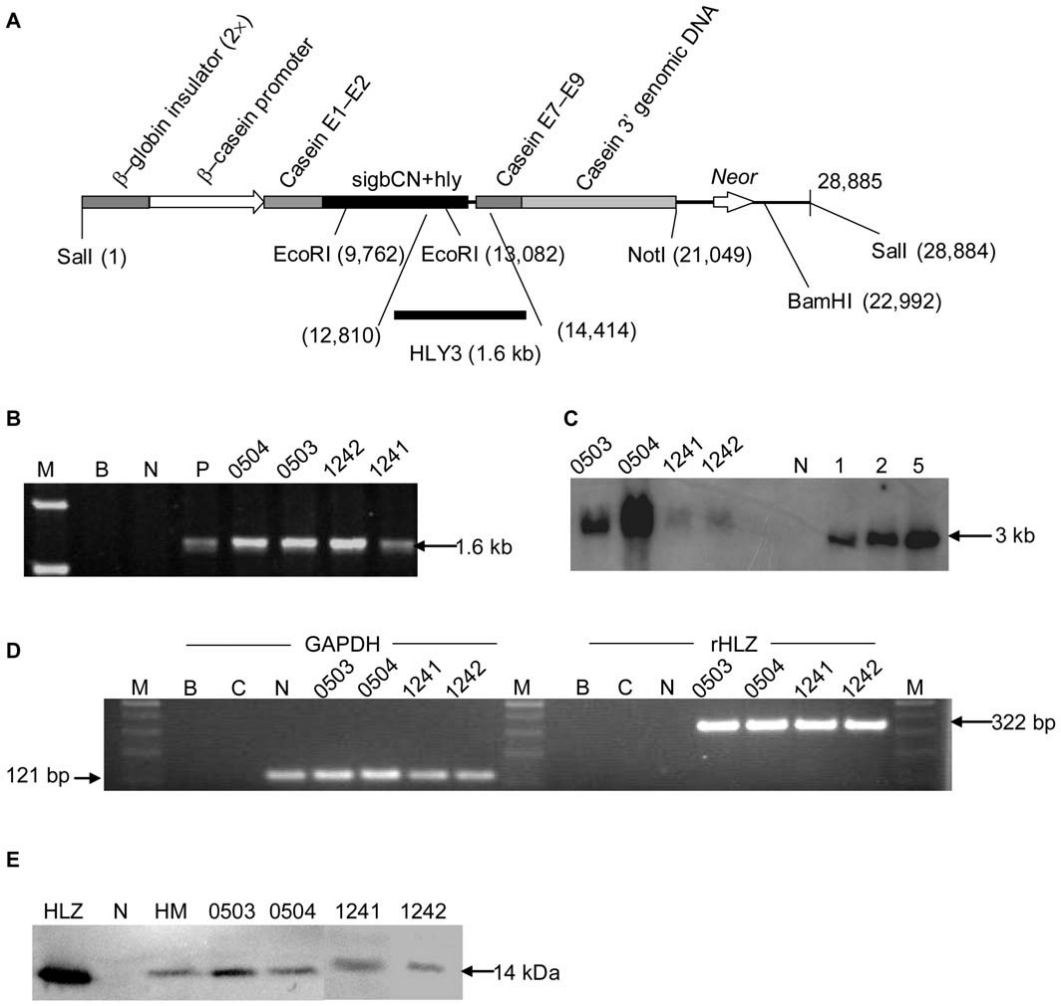
Expression of rHLZ. (A) Schematic drawing of pBC2-HLY-NEOR. The lower bars represent the PCR products used for identification of the transgene. Globin insulator (2×), two copies of chicken β-globin insulator; casein E1–E2, goat β-casein exon 1, intron 1, and part of exon 2 (before initial codon); sigbCN+hly, bovine β-casein signal peptide DNA sequence and HLZ coding region; casein E7–E9, goat β-casein exon 7, intron 7, exon 8, intron 8, and exon 9; casein 3′ genomic DNA, goat β-casein 3′ genomic region. (B) PCR analysis of transgenic cattle. M represents DNA ladder; B represents blank control; N represents non-transgenic cattle and P represents positive control form constructed vector. (C) Southern blot analysis of transgenic cattle. Genomic DNA (20 µg per lane) was isolated from ear biopsies of cattle and digested with EcoRI. The bands were hybridized to a 565 bp specific probe that is complementary to a fragment of the HLZ gene. lanes 1, 2, and 5 represent positive controls with 1, 2, and 5 copies, respectively. N, genomic DNA from a non-transgenic cattle; 0503, 0504, 1241, and 1242 represent genomic DNA from transgenic cattle. (D) RT-PCR analysis of transgenic cattle. Total RNA was isolated from mammary epithelial cells. The 322-bp fragments represent the RT-PCR products of rHLZ, and the 121-bp fragments represent the RT-PCR products of GAPDH. M, DNA ladder; B, blank; C, negative control; N, non-transgenic cattle. (E) Western blot analysis of transgenic cattle. Samples of skim milk (1 µl each) were separated by SDS-PAGE and hybridized with anti-HLZ. HLZ, commercial HLZ (positive control); N, non-transgenic milk; HM, skim human milk; the other lanes, milk from transgenic cattle.

The pBC2-HLY-NEOR transgene vector contains the *HLZ* coding region, a bovine β-casein signal peptide DNA sequence, and one selection marker, the neomycin resistance gene (Neor) were used (Figure 1A). After somatic cell nuclear transfer (SCNT), 312 blastocysts were transferred into recipient cattle. Thirty-seven calves were born at full term (2 from cell strain of fetal genital ridge, FG; 11 from cell strain of fetal oviduct epithelial cells, FOV; 23 from cell strain of fetal oviduct epithelial cells 19, FOV-19; and 1 from cell strain of bovine fetal fibroblasts b1, BWFF-b1). Seven calves died within a few hours after birth, and six calves died within 6 months after birth. Twenty-four calves survived and were healthy after weaning; these calves were from two cell types, genital ridge cells (2 calves) and oviduct epithelial cells (22 calves). After the PCR and Western blot detection, we have created 17 healthy cloned transgenic cattle that expressed rHLZ, but only 4 transgenic cattle, 0503, 0504, 1241, and 1242 were lactating normally in the research time. Thus the data from subsequent experiments to be report just focus on those four transgenic cattle.

By PCR analysis, *HLZ* was integrated into the genome of the four transgenic cattle (Figure 1B). Southern blot analysis revealed that the cattle derived from different cell strains (0503 and 0504 from FOV, 1241 and 1242 from FG) carried different copies of *HLZ* (The copy number of 0503, 0504, 1241 and 1242 are 2, 7, 1, 1 respectively) in their genomes (Figure 1C). RNA expression of *HLZ* was detected in the mammary epithelial cells from each transgenic cow (Figure 1D). Milk from the four transgenic cattle that were lactating normally was collected to confirm the expression of the rHLZ protein. The results showed that all four cattle expressed rHLZ protein (Figure 1E). Radioimmunoassay was used to quantify rHLZ levels. The concentration of rHLZ was 17.69±7.56 µg/ml for cow 1242, 13.25±0.46 µg/ml for cow 1241, 18.99±3.78 µg/ml for cow 0503, and 25.96±2.53 µg/ml for cow 0504. We also detected changes in the concentration of rHLZ in the milk of naturally lactating cow 1242 over the first month of lactation. The amount of rHLZ declined in the first month of lactation but then appeared to stabilize (Figure S1).

### Compositional analysis of the milk derived from transgenic cattle

Milk samples from transgenic and non-transgenic cattle were collected each month for 6 months during their natural lactation period. There were no significant differences on the amount of fat, protein, lactose, and milk solids in the milk of transgenic and non transgenic cattle (P = 0.546, 0.678, 0.672, 0.837, respectively), as shown in Table 1.

**Table 1:** Raw components of transgenic milk compared with conventional milk.

	Transgenic (n = 4)	Non transgenic (n = 4)
Fat (g/100 ml)	4.82±0.67	4.5±0.74
Protein (g/100 ml)	3.32±0.34	3.41±0.23
Lactose (g/100 ml)	4.81±0.08	4.89±0.32
Solids (g/100 ml)	13.65±1.05	13.5±0.98

No significant differences were detected between transgenic group and non transgenic group (P>0.05).

### Purification of rHLZ from transgenic milk

**Figure 2:**
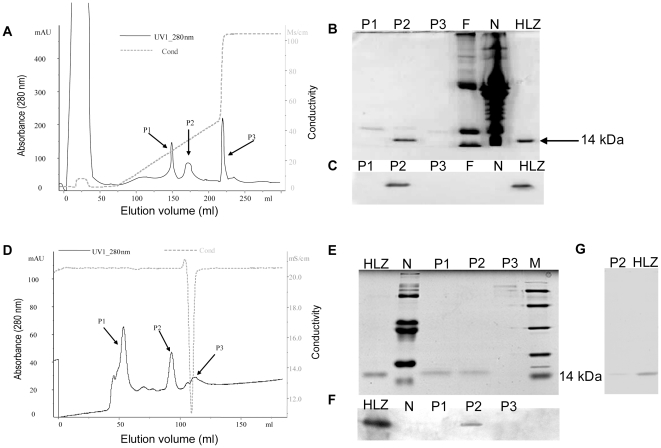
Purification of rHLZ by cation-exchange chromatography and gel filtration chromatography. (A) Purification of rHLZ by cation-exchange chromatography. Transgenic milk whey (15 ml) was diluted in 20 mM sodium phosphate buffer, pH 8.5, and applied to the column. (B) The identification of rHLZ by 15% SDS-PAGE and Coomassie blue staining of the cation-exchange chromatography elution peaks. (C) Western blot identification of the elution peaks after cation-exchange chromatography. HLZ, commercial HLZ; N, milk from a non-transgenic cow; P1, P2 and P3 represent different elution peaks form cation-exchange chromatography; F represents the flow through. (D) Gel-filtration chromatography of the partly purified rHLZ from cation-exchange chromatography. The P2 fraction shown in A was loaded on the column after being concentrated with a centrifugal filter. (E) The identification of rHLZ by SDS-PAGE (15% gel) after Coomassie blue staining of elution peaks. HLZ, commercial HLZ; N, milk from a non-transgenic cow; M, protein marker; P1, P2 and P3 represent different elution peaks form Gel-filtration chromatography. (F) Western blot identification of SP Sepharose elution peaks. HLZ, commercial HLZ; N, milk from a non-transgenic cow; M, protein marker. P1, P2 and P3 represent different elution peaks form Gel-filtration chromatography. (G) The purified rHLZ protein after a two-step purification process was identified by SDS-PAGE (15% gel) and silver staining. HLZ, commercial HLZ. P2 represent elution peaks form Gel-filtration chromatography which is labeled in figure part D.

A two-step purification method was developed to purify rHLZ. The main factors influencing rHLZ extraction are pH value and ionic strength of the buffer [29]. We conducted a series of preliminary experiments to find the best conditions for purification of rHLZ. The results of those experiments showed that the optimal condition was with 20 mM sodium phosphate buffer at a pH of 8.5. As this condition, the elution profile showed the best elution peak width and peak intensity. After removing fat and casein from the milk, a cation exchange column was used during the first step of the purification method to remove most of the whey protein. Most proteins appeared in the elution fluid. rHLZ was eluted with 0.3 M sodium chloride in 20 mM sodium phosphate buffer, pH 8.5 (Figure 2A). SDS-PAGE and western blot analysis determined that the purified protein in peak P2 was rHLZ (Figure 2A, B and C). In order to achieve higher purity (>95%), the fractions containing rHLZ were further purified in the second step of the purification process by gel-filtration chromatography in 0.15 M sodium chloride with 20 mM sodium phosphate buffer, pH 7.2. The high-purity rHLZ in fraction P2 was detected by SDS-PAGE, silver-stained PAGE (15% gel) and western blot (Figure 2D, E, F and G). The purity of the final concentrated product exceeded 95% when analyzed using Quantity One software (Figure 2G). As a control, we also used the same purification procedures to successfully purify HLZ from human milk (data not shown). The highly pure rHLZ was used in subsequent experiments, with commercial HLZ used as a positive control.

### Comparison of the properties of rHLZ with that of other lysozymes

#### Peptide mass fingerprinting, molecular mass, and N-terminal analysis

The bands corresponding to purified rHLZ and commercial HLZ were removed from the gel for peptide mass fingerprinting (PMF). The results showed that purified rHLZ and commercial HLZ have the same fingerprinting patterns, and rHLZ was assigned to HLZ (number of matched peptides and score not shown). SDS-PAGE analysis of purified rHLZ indicated that its apparent mass was similar to that of commercial HLZ (Figure 2G). To improve the accuracy of the mass measurements, we compared the two mass spectra generated by matrix-assisted laser desorption/ionization time-of-flight mass spectrometry (MALDI-TOF MS). The slight molecular mass differences (about 5 Da) between rHLZ and commercial HLZ was detected (Table 2). N-terminal amino acid sequence analysis further confirmed that purified rHLZ was identical to HLZ from breast milk (KVFERCELARTLKRL, GenBank accession no. CAA32175.1; Table 2). rHLZ differed from bovine lysozyme at three amino acids in the N-terminal sequence (K(K)F(Q)RCELARTLK(K)L, GenBank accession no. AAT92538.1).

**Table 2:** Comparison of the protein characteristics of purified rHLZ and commercial HLZ.

	rHLZ from transgenic cattle	Commercial HLZ
N-terminal sequence	KVFERCELARTLKRL	KVFERCELARTLKRL
Molecular mass	14,679.14 Da	14,674.19 Da
Lytic activity (U/mg)	117,089.3±9,471.50	114,413.8±22,470.78

#### Lysozyme activity assay

We used two methods to analyze the bactericidal activity of purified rHLZ, and we compared the activity of rHLZ with that of commercial HLZ and commercial hen egg white lysozyme.

**Figure 3:**
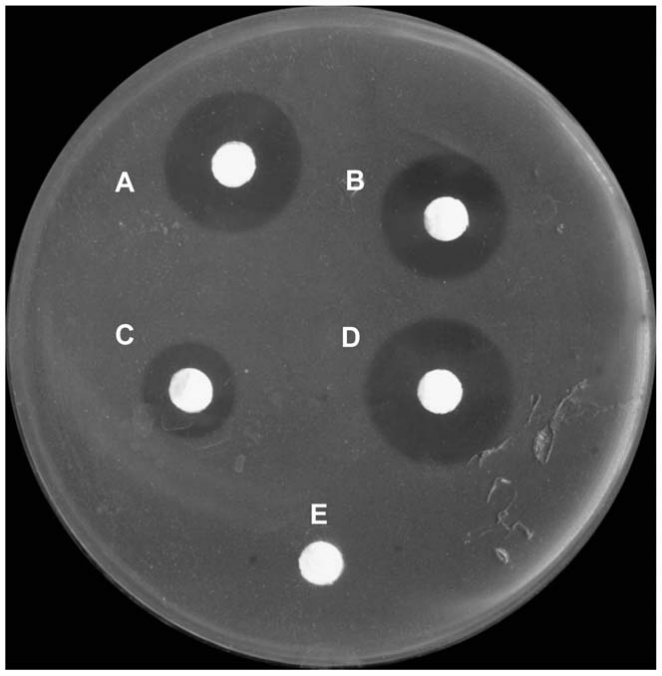
Lytic activity of rHLZ, HLZ, and hen egg white lysozyme against *Micrococcus lysodeikticus*. The small circles (7 mm in diameter) consist of quantitative filter paper with 2 µg of test sample at 0.2 mg/ml or sterile water (control). The larger circles are the inhibition zones. A, commercial HLZ; B, purified rHLZ-1, which was not desalinated; C, hen egg white lysozyme; D, purified rHLZ; E, sterile water (control).

In an agar diffusion test, the inhibition zones for 2-µg samples were clearly visible after an incubation of 24 h. There was no inhibition zones on filter paper filled with distilled water (negative control). rHLZ showed strong lytic activity, similar to that of commercial HLZ. Commercial hen egg white lysozyme had about one-third of the lytic activity of rHLZ (Figure 3). To test the influence of salt concentration on lysozyme activity, we compared the lytic activity of purified rHLZ that had been desalinated with ddH_2_O, termed rHLZ-1, with that of purified rHLZ that had not been desalinated. The lytic activity of rHLZ-1 was lower than that of rHLZ (Figure 3 and Table 3).

**Table 3:** The lytic activity, determined by the turbidimetric method, against *Micrococcus lysodeikticus* cells.

	Concentration (mg/ml)^1^	Activity (mean ± S.D., U/mg)
Commercial HLZ	0.901	114,413.8±22,470.78
rHLZ	0.1966	117,089.3±9,471.50
rHLZ-1^2^	0.1202	79,444.44±3,367.88
HLZ^3^	0.04832	141,979±21,891.54
Hew-LZ^4^	86.3764	42,604.25±5,657.47

1 The concentration of HLZ and rHLZ was quantified by ELISA. The commercial hew-LZ was quantified by UV specrophotometer.

2 rHLZ-1 is purified rHLZ that was not desalinated.

3 HLZ is purified lysozyme from human milk that was purified using the same methodology used to purify rHLZ.

4 Hew-LZ is hen egg white lysozyme.

The same samples also were examined by the turbidimetric method. The data showed that the lytic activity of rHLZ against *Micrococcus lysodeikticus* was similar to that of purified HLZ and two to three times higher than that of commercial hen egg white lysozyme (Table 3). These results were similar to those from the agar diffusion test.

#### Optimal temperature and thermostability

**Figure 4:**
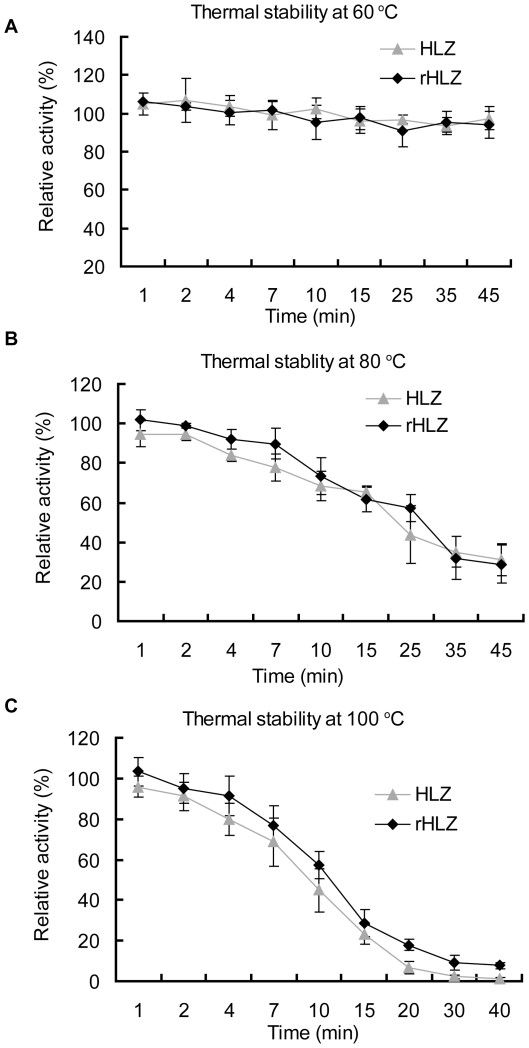
Thermostability of purified rHLZ and HLZ at 60°C, 80°C, and 100°C. The lysozyme activity assay was carried out using the turbidimetric method with a spectrophotometer at *A*_450_. Lytic activity was measured against *Micrococcus lysodeikticus* in 66 mM potassium phosphate buffer, pH 6.24, at 25°C after incubation for different times at 60°C (A), 80°C (B), and 100°C (C). Lysozyme activity measured at 25°C represented 100% activity. rHLZ, purified rHLZ; HLZ, commercial HLZ. The experiment for each group was repeated at least three times, and the results represent mean ± S.D.

From the data shown (Table 4), there were no significant differences between the activities of HLZ and rHLZ in different temperature conditions we set (P>0.05). In addition, the average activity of 40°C was higher than that of 25°C (P = 0.048),60°C (P = 0.006) and 80°C (P = 0.002). And, the different of 25°C and 60°C was not significant (P = 0.247). The relative activity of rHLZ and HLZ were also compared under three different heating conditions (60°C, 80°C, and 100°C) to reveal their super thermostablilty (Figure 4). rHLZ maintained nearly 95% of its activity at 25°C after a 45-min incubation at 60°C. The relative lytic activity of rHLZ and HLZ decreased when incubated at 80°C or 100°C within 40 min. After 10-min incubation at 100°C, the lytic activity was nearly 50% lower than that at 25°C. And lytic activity was very low after a 30-min treatment. The thermostability profiles of rHLZ and HLZ were similar (Figure 4).

**Table 4:** Optimal temperature for purified rHLZ and HLZ by detecting the lysozyme activity.

	Commercial HLZ	rHLZ
25°C (units/mg)	10,445±8,326	120,027±10,035
40°C (units/mg)	121,907±10,006	137,358±3,512
60°C (units/mg)	101,263±7,013	110,007±7,964
80°C (units/mg)	78,750±7,563	86,423±12,550

#### Optimal pH and pH stability

**Figure 5:**
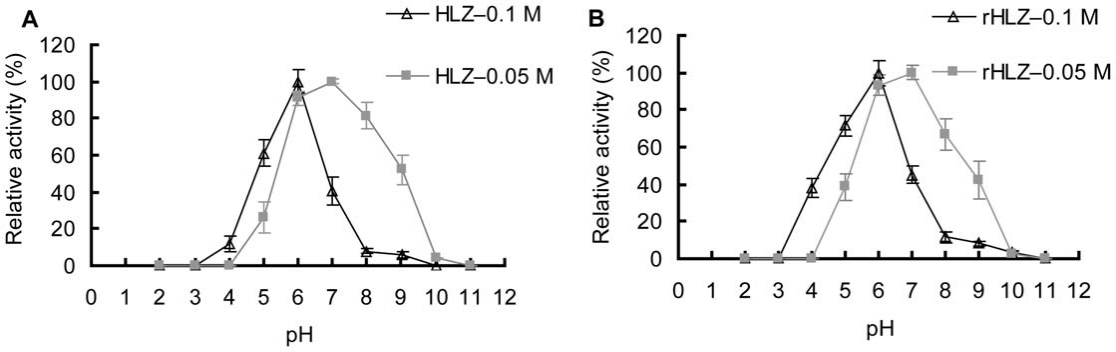
The optimal pH for the lytic activity of purified rHLZ and commercial HLZ in buffers with different salt concentration. The lysozyme activity was measured using the turbidimetric method with a spectrophotometer at *A*_450_. The lytic activity was measured against *Micrococcus lysodeikticus* in sodium acetate buffers of pH 2–5, potassium dihydrogen phosphate buffers of pH 6–8, and carbonate bicarbonate buffers of pH 9–11. Lytic activity measured in potassium dihydrogen phosphate buffer, pH 6, represented 100% activity for salt concentration at 0.1 M. Lytic activity measured in potassium dihydrogen phosphate buffer, pH 7, represented 100% activity for salt concentration at 0.05 M. (A) The optimal pH for commercial HLZ at salt concentration of 0.1 M and 0.05 M. (B) The optimal pH for purified rHLZ at salt concentration of 0.1 M and 0.05 M. The experiment for each group was repeated at least three times, and the results represent mean ± S.D.

**Figure 6:**
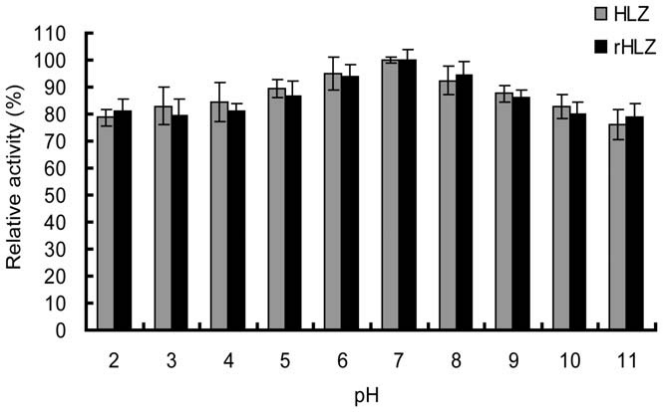
pH stability of purified rHLZ and commercial HLZ. Commercial HLZ and purified rHLZ were incubated in sodium acetate buffers of pH 2–5, potassium dihydrogen phosphate buffers of pH 6–8, and carbonate bicarbonate buffers of pH 9–11 for 20 min. The lysozyme activity was then assayed, using the turbidimetric method, against *Micrococcus lysodeikticus* in potassium dihydrogen phosphate buffer, pH 7, at salt concentration of 0.05 M. The lytic activity incubated in potassium dihydrogen phosphate buffer, pH 7, at salt concentration of 0.05 M represented 100% activity. HLZ represents commercial HLZ; rHLZ represents purified rHLZ. The experiment for each group was repeated at least three times, and the results represent mean ± S.D.

The lytic activity of rHLZ and commercial HLZ was tested using three buffers with different salt concentration at different pH values (pH 2–11). The optimal pH of rHLZ varied with salt concentration of the buffer (Figure 5). The highest activity of the purified rHLZ was at pH 7 when the salt concentration was 0.05 M of the buffer. The highest activity of rHLZ was at pH 6 when the salt concentration of the buffer was 0.1 M. When using a buffer with a salt concentration of 0.05 M, rHLZ showed lytic activity across a broader range of pH values (pH 5–9) than salt concentration of buffer was 0.1 M. The lytic activity of rHLZ declined sharply at extreme pH values in both ionic-strength buffers. Those results were similar to that of HLZ (P>0.05, Figure 5). The data of pH stability revealed the purified rHLZ was quite stable (Figure 6). It also showed that the rHLZ and HLZ were similar in their stability for pH values ranging from 2.0 to 11.0 (P>0.05), indicating that the enzymes are resistant to acid-base environments.

## Discussion

To our knowledge, this is the first study that resulted in the production of a herd of cloned transgenic cattle that expressed rHLZ in their milk. The transgenic milk allows for the transfer of the nutritional aspects of HLZ from human milk to bovine milk. It is fulfilled the conception of humanizing the bovine milk. In addition, a previous report showed that over-expression of antimicrobial compounds in livestock milk can improve udder health and inhibit the microorganisms that cause mastitis [25], [30]; therefore, our model can be explored whether the additional benefits for cattle would exist. Moreover, we have provided a way to produce HLZ, one that can be expanded on an industrial scale.

The modified bovine milk is a possible substitute for human milk which has been shown to be able to produce a number of transgene-expressed proteins [24], [31]. In preliminary work, we have established two different kinds of transgenic mice, pBC-hLY and pBC-sighLY, as model systems for the expression of rHLZ. The highest concentration is about three times than that of the level in human whey. It suggested that the vectors were successfully constructed to express the rHLZ in mammary gland [28]. On the basis of those findings, we modified the pBC-sighLY expression vector to create the pBC2-HLY-NEOR expression vector to promote the expression of rHLZ in transgenic cattle. In milk from four naturally lactating transgenic cattle, the average expression level of rHLZ was lower (13∼26 µg/ml) than other reported recombinant proteins in cattle (lactoferrin) and goats (lysozyme) [27], [32]. Some reasons might cause this difference. Firstly, more regulatory elements in transgenic vector may lead to high expression level, for example using bacterial artificial chromosome (BAC) in other report [32]. Secondly, the different levels of expression among transgenic cattle may relate to the copy numbers of the transgene [33]. Thirdly, transgene may integrate into the chromosome of an inactive transcription region, so a positional effect could cause the expression low [31], [34]. In addition, many factors of the SCNT procedure, such as the quality of the donor cells, the nuclear reprogramming, could also lead to different levels of expression [35]–[37]. The exact mechanisms remain to be determined, however.

To extract rHLZ of high purity (>95%), we established a simple two-step method for the purification of the recombinant protein from milk. This purification scheme provides a new, cost-effective method for the extraction of rHLZ from transgenic milk. Affinity chromatography for the separation of HLZ can yield a high recovery and concentration [38]; however, the media used in affinity chromatography are costly, and the coupling procedure is complex and time consuming. This method therefore has limited application on an industrial scale. Cation-exchange chromatography has advantages over affinity chromatography. It used to be as a common method for the purification of rHLZ in other expression systems [29], [39]. We selected phosphate buffers for purification, and we optimized the pH and salt concentration. The best elution condition was with a buffer of 0.3 M sodium chloride and 20 mM sodium phosphate at a pH of 8.5. Because bovine milk contains only 0.1 µg/ml of lysozyme [16], we are not concerned about the influence of the endogenous lysozyme.

The transgenic technique is associated with the problem of random integration [31], [34]. Incomplete reprogramming of transgenic animals can result in errors in gene expression [40]. Although significant problems still remain, many transgenic animals behave normally and do not show any differences from non-transgenic animals, except for the expression of heterologous protein [32], [41], [42]. Our study provided that the gross composition of milk showed no significant difference between transgenic and non-transgenic cattle (P>0.05). Though the fatty acid and total milk solids of one of the transgenic cattle was a little higher than that of the others, the difference is within the normal range for Holstein cattle as a whole when examining data from the literature [42]–[44]. The pattern of the protein distribution in milk from transgenic and non-transgenic cattle was similar. This conclusion was reached from the results of the SDS-PAGE analysis, but the results will need to be validated by two-dimensional gel electrophoresis, mass spectrometry, and bioinformatics. In addition, the fatty acid, amino acid, mineral, and vitamin composition of the milk produced by transgenic and non-transgenic cattle will need to be measured and validated in future research. Of cause, the health and welfare of the transgenic animals should be considered [45].

We examined the physical and chemical characteristics of the rHLZ secreted in bovine milk. All of the results indicated that the purified rHLZ was similar to the natural HLZ. Although there was a minor difference in molecular mass between rHLZ and HLZ, post-translational modification did not occur with rHLZ. The minimum mass increase of protein peptide levels after different types of post-translational modification is due to methylation with an increase of 14 Da [46]. Peptide molecular fingerprinting maps of purified rHLZ were similar to those of HLZ, which demonstrates that rHLZ has the same primary structure as HLZ. Furthermore, the sequence-determined N terminus of the purified rHLZ in this study matched that of HLZ from another study [47]. This result indicates that the signal peptide of bovine β-casein connecting to rHLZ was cut correctly in the bovine mammary epithelial cells.

A well-known property of lysozyme is its lytic activity against Gram-positive bacteria. rHLZ displayed a level of lytic activity that was similar to that of HLZ (P>0.05) and three times higher than that of hen egg white lysozyme.

The optimal temperature and thermostability of the transgenic milk were assayed because post-processing stability and storage of transgenic milk are important considerations for commercial production. The U.S. Food and Drug Administration requires that Grade A pasteurized milk undergoes a minimum heating at 63°C for 30 min or 72°C for 15 s [48]. For transgenic milk to be processed into milk powder, much higher temperatures (70°C–100°C) must be tolerated during the spray-drying process. In our study, rHLZ purified from transgenic milk had the same optimal temperature and was equally thermostable as HLZ (P>0.05). We conclude that post-processing procedures will have only minor effects on rHLZ activity.

The optimal pH of the purified rHLZ and HLZ varied with salt concentration in this study. A similar phenomenon was observed with hen egg white lysozyme [49] and oyster lysozyme [50]. We also found that the lytic activity of the lysozymes varied with pH. This might be because the different solutions change the positive charge at the surface of the lysozyme and the negative charge of the cell wall, which reacts with lysozyme [51], [52]. The pH stability assay revealed that rHLZ and HLZ were both stable at an acid pH and an alkaline pH (pH 2–11). These properties are similar to those reported by Chandan et al. [16]. The pH of cow milk is around 6.8 [53], which is optimal for the lytic activity of rHLZ. Earlier reports suggest that rHLZ may extend the shelf-life of goat milk [25]. We infer that rHLZ will prolong the shelf-life of transgenic bovine milk, and we are currently studying this possibility.

In conclusion, we successfully produced healthy transgenic cattle that expressed rHLZ. This approach could provide an inexpensive and industrial-scale method for the purification of rHLZ. In addition, we have shown that the enzymatic properties and physicochemical characteristics of rHLZ were identical to those of HLZ. Transgenic cow milk will likely be beneficial to the health of livestock as well, as suggested by a feeding experiment using HLZ-transgenic goat milk [25], [54], [55].

## Materials and Methods

### Production of transgenic cattle

The rHLZ expression vector pBC2-HLY-NEOR (Figure 1A) was derived from the pBC-sighLY expression vector that was expressed previously in transgenic mice [28]. A 31-bp sequence from bp 6,407 to bp 6,438 (GAATTCATTTCCTAATCATGCAGATTTCTAG) of the goat ß-casein promoter was removed from the pBC-sighLY expression vector. A selection marker, *Neor* (neomycin resistance gene), was added to the vector. The new expression vector therefore contained: the *HLZ* coding region; the bovine ß-casein signal peptide DNA sequece; the goat ß-casein promoter without the 31-bp fragment; the 3′ genomic sequence, exon 1, intron 1, part of exon 2 (before the initial codon), exon 7, intron 7, exon 8, intron 8, and exon 9 of goat ß-casein; two copies of the chicken ß-globin insulator; and the selection marker, *Neor*.

The restriction enzyme SalI was used to digest a 28-kb fragment containing *HLZ* from the pBC2-HLY-NEOR expression vector (Figure 1A). The fragment was then transfected into embryonic fibroblasts, oviduct epithelial cells, and genital ridge cells using electrotransformation with a DC pulse of 1.2 kV/cm for 1 ms. After selection with 800 µg/ml Geneticin® (G418; Life Technologies, Carlsbad, CA), several positive colonies were isolated. SCNT was then conducting using the methods of Gong et al. [56]. A total of 312 embryos were transferred into recipient Chinese Luxi yellow cattle. All animal procedures were approved by the Institutional Animal Care and Use Committee at the China Agricultural University(ID: SKLAB-B-2010-003).

### Detection of rHLZ

#### Polymerase chain reaction (PCR) analysis

PCR analysis of DNA from the ears of transgenic and non-transgenic cattle was performed with a pairs of primers. The primers, HLY3 and PCR product information are provided in Table 5.

**Table 5:** Primers for PCR analysis.

Primer name	Primer sequence (5′–3′)	The target amplification
HLY3-F	CAGCACTTTGGGAGACCGA	1,600 bp
HLY3-R	CATCTTCAGTATTTTGCCCTC	
GAPDH-F	CGACTTCAACAGCGACACTCAC	121 bp
GAPDH-R	CACCCTGTTGCTGTAGCCAAA	
RT-F	ATCAGCCTAGCAAACTGGAT	322 bp
RT-R	CTCCACAACCTTGAACATAC	

#### Southern blot analysis

Genomic DNA (20 µg) from ear biopsies of transgenic and non-transgenic cattle was digested using EcoRI and separated on a 1% agarose gel before transfer to a Hybond+ membrane (Amersham Pharmacia Biotech, Piscataway, NJ). Southern blot analysis was performed using a 565-bp, HLZ-specific hybridization probe labeled with digoxigenin (DIG) using a PCR DIG Probe Synthesis kit (Roche, South San Francisco, CA). The positive hybridization signal was a ∼3-kb fragment. Genomic DNA from non-transgenic cattle was used as the negative control. The pBC2-HLY-NEOR plasmid DNA was used as the positive control. Copy numbers of the transgene were quantified using Quantity One software (Bio-Rad, Hercules, CA).

#### Reverse transcription-PCR (RT-PCR) analysis

RNA was extracted from the mammary epithelial cells of transgenic and non-transgenic cattle for use in RT-PCR. The extraction procedure involved centrifuging 100-ml samples of milk at 4,000× *g* for 10 min, carefully removing the milk fat and supernatant on the top layer of each tube, resuspending the sediment in 20 ml PBS with 0.5 mM EDTA, and then centrifuging the suspension at 4,000× *g* for 10 min. Each pellet was used immediately for RNA extraction with the RNeasy® Mini kit (QIAGEN, Hilden, Germany). The RNA samples were eluted with RNase-free water and stored at −80°C.

The first strand of cDNA was synthesized using M-MLV reverse transcriptase according to the manufacturer's instructions (Promega, Madison, WI) and was subsequently used as the template for PCR. RT-PCR primers were designed on the basis of the HLZ coding sequences, and the upstream primer was designed across one intron. The primers (GAPDH and RT) are shown in Table 5. The negative control was performed using RNA pool from all samples as PCR template.

#### Western blot analysis

Transgenic and non-transgenic cattle of 6–8 months of age were induced with agent of induction (National Caotan Pharmacy Company, Xi'an China) to produce milk. Milk was collected three times per week for 1 month. Skim milk which was produced by centrifuging at 4,000× *g* for 10 min was separated by 15% SDS-PAGE and electro-transferred onto nitrocellulose membranes (Amersham Pharmacia Biotech) for western blot analysis. Rabbit polyclonal antibody against HLZ (US Biological, Swampscott, MA) and horseradish peroxidase–conjugated goat anti–rabbit IgG (Biodesign, Saco, ME) were used to detect rHLZ. The positive control used in western blot analysis was the commercial HLZ (Sigma, St. Louis, MO). The negative control was non-transgenic milk.

#### Quantification of rHLZ by radioimmunoassay

The amount of rHLZ in the milk of naturally lactating transgenic cattle over the first month of lactation was carried out at the Institute of Atomic Energy Utilization (Beijing, China) for radioimmunoassay (RIA). The experiment for each sample was repeated at least three times, and the results represent mean ± S.D.

### Analysis of milk components

The transgenic cattle 0503, 0504, 1241, and 1242 were selected for analysis of the gross composition of their milk. The non-transgenic cattle 2006, 1019, 020, and 1009 were used as controls. The breeding conditions were identical for the two groups. Milk samples were collected once a month for a period of 6 months after parturition. The percentage (w/vol) of fat, protein, lactose, and solid were determined using a MilkoScan 4000 (Foss, Hillerod, Denmark).

### Purification of rHLZ from transgenic milk

#### Sample preparation

Milk was defatted by centrifugation at 2,600× *g* at 4°C for 10 min. The whey from each sample was ultracentrifuged at 100,000× *g* at 20°C for 1 h, and it was diluted with 100 mM sodium phosphate, pH 8.5, to a final concentration of 20 mM. The sample solution was filtered with a 0.22-µm filter before use.

#### Purification of rHLZ

A two-step purification procedure was used to isolate rHLZ using an ÄKTA purifier 10 system (Amersham Bioscience, Piscataway, NJ). In the first step, a 15-ml sample was loaded onto a HiLoad 16/10 SP Sepharose HP column (GE Healthcare, Uppsala, Sweden). After equilibration with 20 mM sodium phosphate, pH 8.5, bound proteins were eluted with a linear gradient of 0–0.4 M NaCl in 20 mM sodium phosphate buffer, pH 8.5. In the second step, fractions containing rHLZ were concentrated using a 3-kDa-cutoff ultracentrifuge tube (Utra-15; Millipore Amicon, Bedford, MA) and further purified by gel filtration using a HiLoad 16/60 Superdex 75 prep-grade column (GE Healthcare) in 20 mM sodium phosphate buffer with 0.15 M NaCl, pH 7.2. The rHLZ-containing fractions were concentrated and desalted by ultracentrifugation using a 3-kDa-cutoff ultracentrifuge tube (Millipore Amicon Utra-15). The collecting solution was then freeze-dried using Freezone 6 (Labconco, Kansas City, MO). The rHLZ fraction was identified by SDS-PAGE and western blotting. An ELISA kit (BTI, Stoughton, MA) was used to quantify the purified rHLZ, in accordance with the manufacturer's instructions.

### Characterization of rHLZ

#### N-terminal amino acid sequencing of purified rHLZ and HLZ

Purified rHLZ was sent to Shanghai GeneCore Biotechnologies Co. Ltd. (Shanghai, China) for N-terminal amino acid sequence analysis using automatic Edman degradation. Sequence similarity between the N-terminal amino acid sequence of rHLZ and other proteins was analyzed using the BLAST program and the GenBank databases of the National Center for Biotechnology Information.

#### MALDI-TOF-MS analysis of molecular weight of purified rHLZ and HLZ

High-purity rHLZ and HLZ (Sigma-Aldrich) were assayed by MALDI-TOF-MS (Bruker Daltonics, Billerica, MA) using α-cyano-4-hydroxycinnamic acid as a matrix at the linear pattern [57].

#### Peptide mass fingerprinting of purified rHLZ and HLZ

After separation by SDS-PAGE, the protein band of each of purified rHLZ and standard HLZ (Sigma, St. Louis, MO) was excised and destained using 50% acetonitrile and 50 mM NH_4_HCO_3_. The protein was then digested using sequence-grade trypsin (Sigma, St. Louis, MO) for 16 h at 37°C. The supernatant was collected into a clean tube and extracted two times using 0.1% trifluoroacetic acid at 37°C, and the extraction solutions and supernatants were dried in a speed-vac (Eppendorf) to a 5 µl final volume. Sample (1 µl) and 0.5 µl of matrix solution, purified a-cyano-4-hydroxycinnamic acid (Sigma, St. Louis, MO) were added onto the MALDI plate, which was then dried at room temperature. Finally, the samples were analyzed using Autoflex II (Bruker Daltonics, Billerica, MA) in the reflection mode. Peptide mass fingerprinting was performed using MASCOT database. The obtained mass spectra were downloaded to a database for analysis.

#### Lysozyme activity assay by the turbidimetric rate determination method and the agar disc diffusion method

The turbidimetric method was conducted according to the lytic assay described by Shugar [58]. Briefly, *M. lysodeikticu*s (China General Microbiological Culture Collection Center, Beijing) was used as the substrate, and 2.5 ml of substrate suspension (A_600_≈0.7–0.8) was prepared at 25°C with in 66 mM potassium phosphate buffer, pH 6.24. The reaction was initiated immediately after 100 µl of sample (test group) or ddH_2_O (blank group) was added to the substrate solution. Values were recorded at A_450_ every 15 s over a 3-min period. The ΔA_450_ per minute was used as the maximum linear rate for both the test and blank groups. One unit of lysozyme produces a ΔA_450_ of 0.001 per minute. Protein concentrations were calculated as: U/mg protein = (U/ml enzyme)/(mg protein/ml enzyme). All samples were measured in triplicate.

The disc diffusion method was performed with nutrient broth agar (Sigma, St. Louis, MO). *Micrococcus lysodeikticus* (100 µl) at mid-log phase (*A*_600_≈0.6–0.7) was mixed with 20 ml solid culture medium containing 1.5% agar. Each sample (2 µg) was placed on a sterile, quantitative filter paper disc (7 mm in diameter) on a plate, which was incubated for 24 h at 28°C. The results were assessed by inhibition zones around disc paper. Sterile water was placed on the filter paper disc as a negative control. The experiment was repeated three times.

#### Optimal temperature assay

Quantified and purified rHLZ and HLZ (Sigma, St. Louis, MO) were diluted to a 100-µl volume using 66 mM potassium phosphate buffer, pH 6.24. The collected *M. lysodeikticu*s cells were resuspended in the same buffer at mid-log phase (*A*_600_≈0.6–0.8). The samples and substrate solution were incubated at 25°C, 40°C, 60°C, or 80°C for approximately 5 min. The lysozyme activity of the samples was then quickly measured by the turbidimetric method described above, using triplicate samples.

#### Thermostability assay

The thermostability of the quantified and purified rHLZ and HLZ (Sigma, St. Louis, MO) was tested at 60°C, 80°C, and 100°C. Samples were diluted to 100 µl using 66 mM potassium phosphate buffer, pH 6.24, and incubated at the operating temperature. At each temperature, the thermostability of lysozyme was measured at 0 min and after 1, 2, 4, 7, 10, 15, 25, 35, and 45 min of incubation. The incubated samples were quickly mixed with the bacterial suspension, and the turbidimetric method described above was used to measure the lysozyme activity in triplicate samples.

#### pH and salt concentration optima assay

The lytic activity of purified rHLZ and HLZ (Sigma, St. Louis, MO) against *M. lysodeikticu*s was measured at 10 different buffers of varying pH (pH 2.0, 3.0, 4.0, 5.0, 6.0, 7.0, 8.0, 9.0, 10.0, and 11.0) and two salt concentration buffers (0.1 M and 0.05 M). A sodium acetate buffer was used at pH 2.0–5.0, a potassium dihydrogen phosphate buffer was used at pH 6.0–8.0, and a carbonate bicarbonate buffer was prepared at pH 9.0–11.0. We prepared a total of 20 buffer solutions, and these were used to dilute the *M. lysodeikticus* cells and samples. The solutions were pre-incubated at 25°C for 10 min before measuring the lysozyme activity by the turbidimetric method described above, using triplicate samples.

#### pH stability assay

For the pH stability test, the samples were incubated with the different buffers mentioned above for 20 min at 25°C. The lysozyme activity was assayed by using *M. lysodeikticus* in a potassium dihydrogen phosphate buffer, pH 7.0 of 0.05 M, and the turbidimetric method described above, using triplicate samples.

### Statistical analysis

Data for milk components, temperature treatment and pH treatment assay were analyzed by T test using SPSS 13.0, to detect difference between the test group and control group. All values are reported as the mean ± SD.

## Supporting Information

Figure S1 **The expression level of rHLZ in the milk of transgenic cloned cattle 1242 at the first month after lactation.** The concentration of rHLZ was determined by RIA.
(TIF)
