# Supplementary figures and images for: Characterization of Bioactive Recombinant Human Lysozyme Expressed in Milk of Cloned Transgenic Cattle
Source: PLoS One. 2011 Mar 16;6(3):e17593. doi: 10.1371/journal.pone.0017593 (PMC3059212; doi:10.1371/journal.pone.0017593)

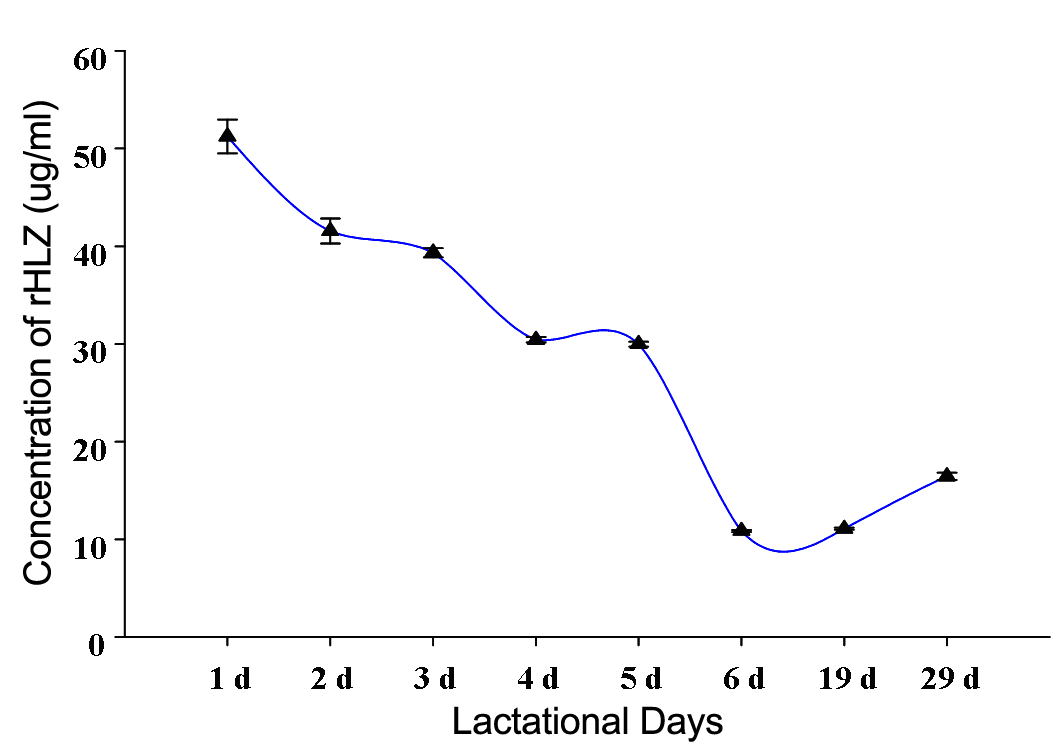

Supplement: Figure S1 — The expression level of rHLZ in the milk of transgenic cloned cattle 1242 at the first month after lactation. The concentration of rHLZ was determined by RIA. (TIF) [file pone.0017593.s001.tif]
